# The Reliability and Validity of the Persian Version of Three-Factor Eating Questionnaire-R18 (TFEQ-R18) in Overweight and Obese Females

**Published:** 2017-04

**Authors:** Seyed-Ali Mostafavi, Shahin Akhondzadeh, Mohammad Reza Mohammadi, Mohammad Reza Eshraghian, Saeed Hosseini, Maryam Chamari, Seyed Ali Keshavarz

**Affiliations:** 1 Psychiatry & Psychology Research Center, Roozbeh Hospital, Tehran University of Medical Sciences, Tehran, Iran.; 2 Department of Biostatistics & Epidemiology, School of Public Health, Tehran University of Medical Sciences, Tehran, Iran.; 3 School of Nutritional Sciences & Dietetics, Tehran University of Medical Sciences, Tehran, Iran.; 4 Obesity & Eating Habits Research Center, Endocrinology and Metabolism Molecular-Cellular Sciences Institute, Tehran University of Medical Sciences, Tehran, Iran.

**Keywords:** *Eating Behavior*, *Obesity*, *Overweight*, *Appetite*, *Three- Factor Eating Questionnaire Reduced-R18 (TFEQ-R18)*, *Iran*, *Females*

## Abstract

**Objective** **:** The Three-Factor Eating Questionnaire Reduced (TFEQ-R18) is one of the most widely used instruments for assessing eating behavior worldwide. The present study aimed at confirming the reliability and validity of the Persian version of TFEQ-R18 among overweight and obese females in Iran.

**Method:** In the present study, 168 overweight and obese females consented to participate. We estimated the anthropometric indices and asked the participants to complete the TFEQ-R18. Beck Depression Inventory (BDI), Spielberger Anxiety Scale, Appetite Visual Analogue Rating Scale, Food Craving Questionnaire (FCQ), Compulsive Eating Scale (CES), and Restraint Eating Visual Analogue Rating Scale were performed simultaneously to assess concurrent validity. Two weeks later, TFEQ-R18 was repeated for 126 participants to assess test-retest reliability. Moreover, we reported the internal consistency and factor analysis of this questionnaire.

**Results:** Using the results of the reliability analysis and exploratory factor analysis of the principal component by varimax rotation, we extracted 3 factors: hunger, cognitive restraint, and emotional eating. After removing the Items 16 and 18, the Cronbach’s alpha was increased to 0.73 (The Cronbach’s alpha of the factors was 0.84, 0.64, and 0.7, respectively). The results of the Pearson correlation revealed a consistency of 0.87 between the test and retest administrations (p = 0.001). Significant positive correlations were observed between TFEQ-R18 and BDI, Spielberger Anxiety Scale, FCQ, CES, appetite, body weight, fat percentage, and calorie intake. Moreover, a negative correlation was observed in Restraint Eating Visual Analogue Rating Scale and muscle percentage**.**

**Conclusion:** This study aimed at presenting preliminary support for the reliability and validity of the Persian version of TFEQ-R18 and its psychometric characteristics. This instrument may be helpful in clinical practice and research studies of obesity, appetite, and eating behavior.

Binge eating disorder is a widespread type of disturbed eating behavior, especially among female dieters (1). Eating behavior and nutritional status are associated with body health and mood (2, 3). Hence, using a reliable and valid instrument to measure its dimensions is of prime importance in clinical practice and research. The Three-Factor Eating Questionnaire (TFEQ), which was first created by Albert J. 

Stunkard (4) is an instrument widely used in eating behavior researches in English speaking population (4). 

This instrument has been translated and validated in many languages and societies including adults and adolescents. Later, Karlsson et al. (5) reported some construction problems with original TFEQ and decided to revise and reduce the items of the questionnaire. The new 18-item questionnaire with 3 subscales was created and named the TFEQ-R18. Eleni Kavazidou et al. 

showed an acceptable psychometric properties of the TFEQ-R18 for 12 to 45 year old males and females in Greek population (6). Loffler, A. et al. performed the confirmatory factor analysis of TFEQ among German population and assessed the association between TFEQ and BMI. They confirmed the following factors: uncontrolled eating, cognitive restraint, and emotional eating. The BMI values were most strongly correlated with uncontrolled eating sub-score (r = 0.26)(7).

Disturbed eating behavior is integrated with psychiatric and appetite disorder and is associated with higher body mass index, (4, 8) and dietary intake of plenty of junk foods (9), and it is more prevalent in females, especially those seeking weight reduction (10). People with disturbed eating behavior usually overeat due to feeling lonely, bored, and depressed (3, 11). They also fail to stop overeating sugary and high fat foods. Hence, this behavior is highly connected with overweight or obesity situations (12). Perhaps, other psychiatric comorbidities occur much more in obese patients with disturbed eating behavior (10). Disturbed eating behavior can be seen in the context of borderline personality disorder, atypical depression, and anxiety. Furthermore, severity of comorbid depression and appetite disorders are associated with the severity of disturbed eating behavior (10).

Valid and reliable instruments are needed in clinical practice and in research to evaluate and interpret subjective measurements such as appetite and eating behavior. Although the aforementioned instrument is reported to be valid and reliable in measuring dimensions of eating behavior in Western societies, due to cultural differences between the Western and Eastern countries, we decided to conduct a confirmatory factor analysis with maximum likelihood and test its reliability and validity in Persian overweight or obese females for the first time.

This study aimed at displaying the association of TFEQ-R18 with some similar questionnaires, body composition, and dietary intake factors (concurrent validity). We also aimed at providing construct validity, internal consistency, test-retest, and factor analysis of the TFEQ-R18 in Iranian overweight and obese females.

## Materials and Methods

***Participants***

The statistical population was overweight and obese females. The participants were 168 overweight and obese females who referred to a weight reduction clinic. They were selected using the convenience sampling method. Inclusion criteria were female gender, being overweight or obese, and signing the consent form. Before starting a weight loss program, the participants were invited to take part in this study. The study protocol was described to them and informed consent was obtained. A clinical dietitian preformed anthropometric assessments including body weight, height, and waist and hip circumferences. Total body fat and muscle percentage were estimated with body composition analyzer all in standard situations. We used Omron HBF-500 BIA (Omron Co., Japan) device, which involved 8 electrodes, tetrapolar electrodes in footpads, and another 4 sets of electrodes in the handle. Each participant stood on the metal footpads in bare feet and grasped a pair of electrodes fixed to a handle with arms extended in front of the chest. This instrument assesses total body fat, visceral fat, lean body mass, and basal metabolic rate as well as body weight and BMI. The clinical validity of this instrument in measuring body composition has already been approved in comparison with Dual-Energy X-Ray Absorptiometry and Magnetic Resonance Imaging (MRI) (13). Then, the participants were asked to fill in the Three-Factor Eating Questionnaire-R18 (TFEQ-R18), Beck Depression Inventory, Spielberger Anxiety Scale, Appetite Visual Analogue Scale, and Compulsive Eating Scale. Furthermore, the participants were asked to fill in a 3-day food record, which was completed at home on 2 nonsequential weekdays, and on 1 day in the weekend, with the days being assigned randomly. The participants were instructed to record everything that they ate or drank including liquids, sweets, and snacks. A guide on portion sizes and scales were also delivered to them. They were required to deliver the 3-day food records 2 weeks later in the second visit. Two weeks later, the tests (TFEQ-R18) were repeated for 126 participants in similar situations.

***Instruments***

-Three-Factor Eating Questionnaire-R18

This questionnaire is able to distinguish among different eating patterns in a general population. Here we want to assess its reliability and validity in overweight or obese women.

-Compulsive Eating Scale (CES)

This instrument was first created by Kagan & Squires. It is an 8-item self-report instrument made to measure the severity of binge eating disorder. Mostafavi and colleagues have validated the Persian version of this tool in Iranian obese individuals (14). Factor analysis of this instrument showed 2 factors: eating because of negative feelings and overeating. The internal consistency of the CES was 0.85. 

-Food Craving Questionnaire (FCQ)

The clinical validity of this instrument was approved in patients with eating disorder. The internal consistency and reliability indexes of the tool ranged from moderate to excellent. This scale could predict the symptoms of eating disorder (15). The factor analysis, and the reliability and validity of the Persian version of FCQ are under study by the current team. 

-Beck Depression Inventory

It is a 21-item self-report questionnaire that assesses symptoms of depression in adults. Stephen Dobston and Parvaneh Mohammad Khani (16) reported an acceptable validity and reliability of the Persian version of the questionnaire among Iranian adult population (Cronbach’s Alfa: 0.92).

***-***Spielberger Anxiety Scale

This instrument is a self-evaluation tool used by many researchers worldwide; its internal consistency is relatively high (Cronbach’s Alfa: 0.90). Panahi et al. have reported the test-retest reliability with correlation coefficient of 0.84 (17).

-Appetite Visual Analogue Scale 

This rating scale is widely used in appetite research. Parker et al. tried to assess the validity of the visual analogue scale and reported a significant correlation between food intake and this tool (18).

-Weight and Height Gage 

A trained dietitian performed all weight and height measurements in standard situations.

-Body Composition Analyzer; Omron HBF-500 BIA (Omron Co., Japan); The validity and reliability of this device was approved by Magnetic Resonance Imaging (MRI) and Dual-Energy X-Ray Absorptiometry (19).

***Procedure***

The TFEQ-R18 was translated into Persian by the first author (SA. M). Then, the third author who is a psychiatrist and native in Persian and fluent in English (MR. M) confirmed the accuracy of the translation and content validity of the Persian version of the TFEQ-R18. An independent translator back translated it into English. The identical content of the 2 versions was confirmed by a language expert. Also, the face and content validity of the questionnaire was checked by all authors (10) and it was good. Next, 200 overweight or obese females were invited and consented to participate in the study; 168 questionnaires were completed by the participants at the first visit, and their data were used for factor analysis and internal consistency measurements; 126 participants completed the study procedure successfully with retest section 2 weeks later, and their data were used for test-retest reliability. 

***Data Analysis***

Descriptive statistics were used to describe the participants, and the Cronbach’s Alpha was used to assess its internal consistency. We performed confirmatory factor analysis to confirm the dimensions and name the factors of the questionnaire. Pearson correlation coefficient was used to assess the test–retest reliability. We also assessed the correlations between TFEQ-R18 and Beck Depression Inventory, Spielberger Anxiety Scale, Appetite Visual Analogue Scale, Food Craving Questionnaire (FCQ), Compulsive Eating Scale (CES), and some anthropometric indices (eg, weight, body fat, and muscle percentages, and waist circumference), and dietary intake of calorie, fat, protein, and sugar. All the analyses were performed using PASW Statistics 18, Release Version 19.0.0 (SPSS, Inc., 2009, Chicago, IL, www.spss.com). Significance level was set at p <0.05.

## Results

Subjects were 18-60 year overweight or obese women with mean age of 39±10.9 year. The demographic characteristics of participants are presented in Table 1.

*Analysis of Internal Consistency of the TFEQ-R18*

Item number 18 was put aside since it was out of the Likert scale. The preliminary reliability of TFEQ-R18 as measured using Cronbach’s alpha coefficient was 0.699 which was very near to acceptable. If the item number 16 was also removed its cronbach’s alpha had increased to 0.73 (Cronbach’s alpha of the factors was 0.84, 0.64, and 0.7, respectively). Overall, the results revealed an acceptable level of internal consistency for the remaining 16 items, and they were homogenous. Hence, items number 16 and 18 did not entered into the factor analysis. 

***Factor Analysis***

We used confirmatory factor analysis to analyze the construction of the questionnaire and name the factors. We benefited from the principal components analysis to extract the factors by varimax rotation. We used the rotation method because we supposed that the factors were correlated with each other, or the components were not independent of each other. Some investigators have proposed that if a factor explains 5% or more of the total variance, that factor is significant (19).

**Figure 1:**
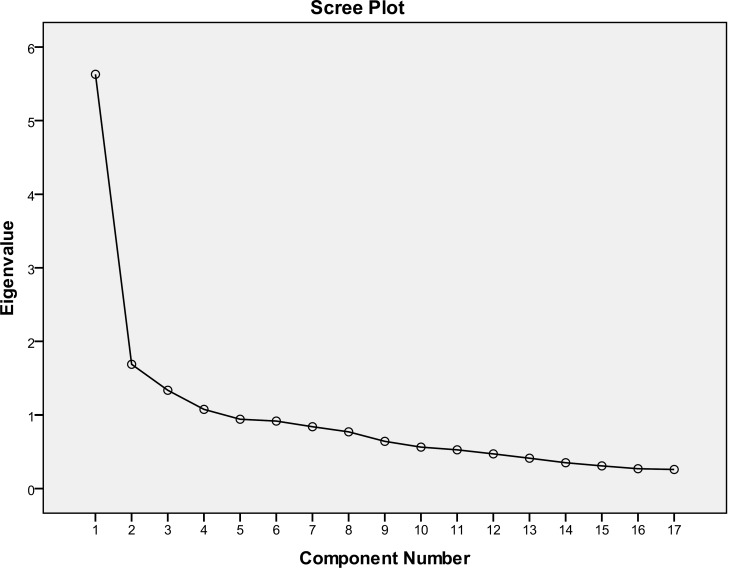
Factors Extracted via Scree Plot

We examined the Kaiser's measure of sampling adequacy to decide whether we could perform factor analysis on our data set and to decide whether any items could be removed. The Kaiser–Meyer–Olkin (KMO) index was 0.83 (Bartlett’s test of sphericity was significant at P<0.0001, df=153). Therefore, the hypothesis of inter-correlation of the variables used in the analysis in the studied population was accepted. Furthermore, based on the Kaiser–Meyer–Olkin (KMO) index, factor analysis was allowed via correlation matrix (Table 2). The range of factor loadings for the items and their variance as well as the Eigen values are displayed in Table 3; Eigen values of greater than 1.00 explained variance of 50.9%. Moreover, factor analysis via scree plot is plotted in Figure 1. Three components were loaded on expected factors. Factor 1 was stronger and explained the greater percentage of the variance (33.1%); 9 items (1, 4, 5, 7, 8, 9, 10, 13, 14) were loaded on this factor (Table 4), and the factor was named hunger. The second factor was named cognitive restraint and explained 9.9 % of the variance (Items 2, 11, 12, 15). The third factor was named emotional eating and explained 7.8% of the variance (Items 3 and 6). The Items 16 and 18 were eliminated from the factor analysis due to lack of consistency with other items of the questionnaire and being out of the Likert scale. The results of the confirmatory factor on 16 items from TFEQ analysis, with varimax rotation, are demonstrated in Table 4.

***Test-Retest Reliability***

The test–retest reliability of the questionnaire was administered to 126 participants in a 2- week interval. The results of the Pearson correlation revealed a consistency of 0.87 between the 2 administrations (p = 0.001).

***Validity of the TFEQ-R-18***

*Concurrent Validity*

The Intercorrelations among TFEQ-R-18 scores, body composition, anthropometric indices, psychometric indices, eating scales, dietary intake of carbohydrates, fat, protein, and calorie are presented in Table 5.

The effect size of the correlation between the TFEQ-R-18 scores and anthropometric indices, psychometric 

questionnaires, and dietary intake provided evidence for the concurrent validity of the TFEQ-R-18.

**Table 1:** Obesity Start Age and Demographic Characteristics of the Participants

	N*	**Percent**
Obesity Start Age: - Before puberty - After puberty	25 143	14.9 85.1
Education: - Diploma or lower - Associate degree or BA - Postgraduate	81 75 12	48.2 44.6 7.1
Socioeconomic Status - Low - Middle - High	19 141 8	11.3 83.9 4.8
Marital Status - Single - Married - Widowed or divorced	26 139 3	15.5 82.7 1.8
Child Delivery Number 0 1 2 3 4 5	35 39 53 35 5 1	20.8 23.2 31.5 20.8 3.0 0.6

**Table 2:** Kaiser–Meyer–Olkin (KMO) Measure of Sampling Adequacy and Bartlett's Test of Sphericity

**Kaiser-Meyer-Olkin (KMO)**		0.836
**Bartlett's Test of Sphericity**	Approx. Chi-Square	987.55
	df.	136
	Sig.	<0.0001

**Table 3:** Eigenvalues and Percentages of the Variance Associated With Each Component

**Component**	**Eigenvalues**	**% of explained Variance**	**Cumulative % of the Explained Variance**
1	5.629	33.112	33.112
2	1.688	9.932	43.044
3	1.335	7.856	50.900

**Table 4:** Rotated Component Matrix^a^ for items of TFEQ-R-18

	**Component**		
	**1**	**2**	**3**
1. When I smell a sizzling steak or juicy piece of meat, I find it very difficult to keep from eating, even if I have just finished a meal.	0.633		
2. I deliberately take small helpings as a means of controlling my weight.		0.602	
3. When I feel anxious, I find myself eating.			0.623
4. Sometimes when I start eating, I just cannot seem to stop.	0.622		
5. Being with someone who is eating often makes me hungry enough to eat also.	0.692		
6. When I feel blue, I often overeat			0.782
7. When I see a real delicacy, I often get so hungry that I have to eat right away.	0.677		
8. I get so hungry that my stomach often seems like a bottomless pit.	0.801		
9. I am always hungry so it is hard for me to stop eating before I finish the food on my plate.	0.542		
10. When I feel lonely, I console myself by eating.	0.528		
11. I consciously hold back at meals in order not to weight gain.		0.682	
12. I do not eat some foods because they make me fat.		0.686	
13. I am always hungry enough to eat at any time.	0.517		-0.519
14. How often do you feel hungry?	0.574		
15. How frequently do you avoid stocking up on tempting foods?		0.701	
16. Is it likely for you to consciously eat less than you want?^b^			
17. Do you go on eating binges when you are not hungry?	0.659		

Extraction Method: Principal Component Analysis

Rotation Method: Varimax with Kaiser Normalization

a.      Rotation converged in 7 iterations.

b.      Item 16 was excluded from the factor analysis due to lack of internal consistency with other items.

**Table 5:** The Intercorrelations Among the TFEQ-R-18 and Other Concurrent Measured Variables for 126 Participants

	**TFEQ-R18**	
	**Pearson Correlation**	**Sig (2-Tailed)**
Body Weight	0.186	0.03
Waist circumference	0.182	0.04
BMI	0.176	0.04
Body Fat%	0.169	0.06
Body Muscle %	-0.111	0.20
Beck Depression Inventory (BDI)	0.30	0.001
Spielberger Anxiety Scale	0.24	0.015
Food Craving Questionnaire	0.47	0.000
Compulsive Eating Scale Appetite Visual Analogue Scale	0.65	0.000
	0.59	0.000
Restraint Eating Visual Analogue Scale (Items 18 of the TFEQ-R18)	-0.54	0.000
Dietary Calorie intake	0.30	0.002
Dietary Carbohydrate intake	0.34	0.000
Dietary Fat intake	-0.025	0.798
Dietary Protein intake	0.074	0.457
Dietary Sugar intake	0.273	0.005

## Discussion

This was the first study to assess the psychometric characteristics of the TEFQ-R18 and report its reliability and validity among Iranian population and in overweight and obese females. We used the confirmatory factor analyses with maximum likelihood because there was preliminary information about the number of factors before the analysis. TEFQ-R18 instrument was made to measures 3 dimensions of human eating behavior (dietary restraint, dietary disinhibition, and hunger) in English population (4). Later, Karlsson et al. reported some construction problems with original TFEQ and decided to revise and reduce the items of the questionnaire. The new 18-item questionnaire with 3 subscales was created and named the TFEQ-R18. They proposed that disinhibition and hunger scales could be grouped in a single subscale and be labeled as uncontrolled eating (UE), with 9 questions. The Cognitive Restraint Scale (CR) was shortened to 6 subscales, and a third subscale was added including 3 items tagged as emotional eating (EE). Similarly, in our study, 3 components were loaded on the expected factors. Factor 1 was stronger and explained the greater percentage of the variance (33.1%); 9 items (1, 4, 5, 7, 8, 9, 10, 13, 14, 17) were loaded on this factor (Table 4), and the factor was named hunger. The second factor was cognitive restraint and explained 9.9 % of the variance (Items 2, 11, 12, and 15). The third factor was named emotional eating and explained 7.8% of the variance (Items 3 and 6). The Items 16 and 18 were eliminated from the factor analysis due to lack of consistency with other items.

Our findings indicated that the preliminary reliability of the TFEQ-R18 measured using Cronbach’s alpha coefficient was 0.699, which was very near to acceptable. However, after removal of Items 16 and 18, the Cronbach’s alpha had increased to 0.73 (The Cronbach’s alpha of the factors was 0.84, 0.64, and 0.7, respectively.). Supornpim Chearskul et al. (20) reported Cronbach’s alpha coefficients of 0.78, 0.75, and .87 for 3 dimensions of the 51- item Thai version of the TFEQ, and the Cronbach’s alpha for the total questionnaire was 0.806. Also, test–retest reliability by Pearson’s correlation was 0.936 (21).

Some studies revealed that nutritional elements affect depression and mood (22-24). Ghiz l. et al. (12) reported that the Three- Factor Eating Questionnaire (TFEQ) had a significant positive correlation with BDI, and Compulsive Eating Scale (CES) in females. Furthermore, Ondercin et al. reported that females with higher scores of disturbed eating behavior more often respond to emotional states such as anxiety, boredom, loneliness, and depression (25). In our study, significant positive correlations were detected among the TFEQ-R18 scores, BDI, Appetite Visual Analogue Scale, Spielberger Anxiety Scale, Food Craving Questionnaire, and Compulsive Eating Scale. Moreover, the negative correlation between the TFEQ and Restraint Eating Visual Analogue Scale (the TFEQ-R18 subscale; r = -0.54) (that we logically expected) demonstrated acceptable convergent and divergent validity. The results of our study revealed that the more anxiety, apprehension, and depression an overweight or obese female feels, the more likely she is to eat emotionally.

In our study, the TFEQ-R-18 was positively correlated with body weight, BMI, waist circumference, and total body fat percentage (convergent validity), but was negatively correlated with muscle percentage (divergent validity; Table 5). In Table 5, the direction of correlation was of greater interest than the power of association. Similarly, Timmerman et al. (26) examined the correlation between disturbed eating, caloric intake, body fat percentage, and BMI in nonpurge binge eating females. They reported that there was a weak, but significant relationship between the severity of disturbed eating behavior and BMI; however, the relationship between the severity of disturbed eating behavior and body fat percentage was not significant. In addition, other researchers revealed a relationship between anthropometric indices and mood and eating disorders (3, 11). Supornpim Chearskul et al. (20) also reported positive correlations between TFEQ factors and BMI (r = 0.17) and body fat percentage (r = 0.32). Charlotte J. Harden et al. reported that overweight participants had significantly higher hunger and disinhibition scores (TFEQ subscales) compared with their normal weight controls. Provencher et al. (27) studied the correlation of eating behaviors and body composition indexes in males and females from the Québec Family Study and reported significant positive correlations between BMI and susceptibility to hunger and BMI and disinhibition in both males and females. A. Lesdéma et al. (28) also reported a positive correlation between TFEQ and BMI. All these reports are in line with our results.

Furthermore, in our study, TFEQ was positively correlated with the dietary intake of energy (r = 0.3), protein (r = 0.07), carbohydrates (r = 0.34), and simple sugar (r = 0.27), but not fat (negative relationship; r = -0.025). This finding is controversial because some sources have reported higher amount of sugary and high fat foods consumption in binge eaters (10). However, others have reported that sugar and fat act differently on the brain receptors, especially pleasure and reward regions, which are associated with eating behavior. Stice et al. (29) reported that sugar may more vigorously influence eating behavior than fat. Furthermore, Yu, Z. et al. reported that in rat models of binge eating, ovarian hormones restrain the fat intake (30). In addition, because these obese females are selected along with a weight reduction diet program, they might have reduced their fat intake prior to the study. Likewise, Blandine de Lauzon et al. (31) reported that the higher the cognitive restraint, the lower the contribution of fat to dietary energy consumption (negative correlation). Altogether, these findings revealed good concurrent and convergent validity of TFEQ-R18.

## Limitations

A limitation of this study was that the study population was limited to females. Hence, we suggest repeating a validation study among males and children or the elderly population.

## Conclusion

The present study revealed that the Persian version of the TFEQ-R18 is a psychometrically sound instrument to assess eating psychopathology in Iranian overweight and obese females. However, the results should be interpreted with caution. The nature of the sample (ie, only the clinical sample of the overweight and obese females) limited the generalizability of the findings into the general population and males.

## References

[1] Grilo CM (2006). Cognitive behavioural therapy does not improve outcome in obese females with binge eating disorder receiving a comprehensive very low calorie diet programme. Evidence-based mental health.

[2] Christensen L (1993). Effects of eating behavior on mood: a review of the literature. The International journal of eating disorders.

[3] Ahmadi SM, Keshavarzi S, Mostafavi SA, Bagheri Lankarani K (2015). Depression and Obesity/Overweight Association in Elderly Females: a Community-Based Case-Control Study. Acta medica Iranica.

[4] Stunkard AJ, Messick S (1985). The three-factor eating questionnaire to measure dietary restraint, disinhibition and hunger. Journal of psychosomatic research.

[5] Karlsson J, Persson LO, Sjostrom L, Sullivan M (2000). Psychometric properties and factor structure of the Three-Factor Eating Questionnaire (TFEQ) in obese men and females. Results from the Swedish Obese Subjects (SOS) study. Int J Obes Relat Metab Disord.

[6] Eleni Kavazidou Mp, Ioannis Liolios, George Doganis, Katerina Petrou, Agathoklis Tsatsoulis, Anna Tsiligiroglou-Fachantidou (2012). Structure validity of the Three-Factor Eating Questionnaire-R18 in Greek population. Journal Of Human Sport & Exercise.

[7] Loffler A, Luck T, Then FS, Sikorski C, Kovacs P, Bottcher Y (2015). Eating Behaviour in the General Population: An Analysis of the Factor Structure of the German Version of the Three-Factor-Eating-Questionnaire (TFEQ) and Its Association with the Body Mass Index. PloS one.

[8] Atlas JG, Smith GT, Hohlstein LA, McCarthy DM, Kroll LS (2002). Similarities and differences between Caucasian and African American college females on eating and dieting expectancies, bulimic symptoms, dietary restraint, and disinhibition. The International journal of eating disorders.

[9] Harden CJ, Corfe BM, Richardson JC, Dettmar PW, Paxman JR (2009). Body mass index and age affect Three-Factor Eating Questionnaire scores in male subjects. Nutrition research.

[10] American Psychiatric Association (2013). Binge Eating Disorder, DSM-5 Diagnostic Criteria. Diagnostic and Statistical Manual of Mental Disorders.

[11] Ahmadi SM, Mohammadi MR, Mostafavi SA, Keshavarzi S, Kooshesh SM, Joulaei H (2013). Dependence of the geriatric depression on nutritional status and anthropometric indices in elderly population. Iranian journal of psychiatry.

[12] Ghiz L, Chrisler JC (1995). Compulsive eating, obsessive thoughts of food, and their relation to assertiveness and depression in females. Journal of clinical psychology.

[13] Mock M, Ryan ED, Gerstner GR, Tweedell AJ, Kleinberg CR, Hirsch KR (2016). Validity of a Multi-compartment Body Composition Model Using Body Volume Derived from Dual-Energy X-ray Absorptiometry. Medicine and science in sports and exercise.

[14] Mostafavi SA, Keshavarz SA, Mohammadi MR, Hosseini S, Eshraghian MR, Hosseinzadeh P (2016). Reliability and Validity of the Persian Version of Compulsive Eating Scale (CES) in Overweight or Obese Females and Its Relationship with Some Body Composition and Dietary Intake Variables. Iranian journal of psychiatry.

[15] Moreno S, Rodriguez S, Fernandez MC, Tamez J, Cepeda-Benito A (2008). Clinical validation of the trait and state versions of the Food Craving Questionnaire. Assessment.

[16] Khani kSDPM (1389). [Psychometric propeties of beck depression inventory II in a sample of adults with major depressive disorder (persion)]. Tavanbakhshi.

[17] Panahi (1372). [Preliminary study of reliability and validity of Spielberger trait anxiety inventory (persian)].

[18] Parker BA, Sturm K, MacIntosh CG, Feinle C, Horowitz M, Chapman IM (2004). Relation between food intake and visual analogue scale ratings of appetite and other sensations in healthy older and young subjects. European journal of clinical nutrition.

[19] Bosy-Westphal A, Later W, Hitze B, Sato T, Kossel E, Gluer CC (2008). Accuracy of bioelectrical impedance consumer devices for measurement of body composition in comparison to whole body magnetic resonance imaging and dual X-ray absorptiometry. Obesity facts.

[20] Supornpim Chearskul SP, Siriporn Vongsaiyat, Patriya Janyachailert, Sucheera Phattharayuttawat (2010). Thai version of Three-Factor Eating Questionnaire. Appetite.

[21] Ignacio Jáuregui-Lobera PG-C, Rocío Carbonero-Carreño, Alejandro Magallares, Inmaculada Ruiz-Prieto (2014). Psychometric Properties of Spanish Version of the Three-Factor Eating Questionnaire-R18 (Tfeq-Sp) and Its Relationship with Some Eating- and Body Image-Related Variables. nutrients.

[22] Ranjbar E, Kasaei MS, Mohammad-Shirazi M, Nasrollahzadeh J, Rashidkhani B, Shams J (2013). Effects of zinc supplementation in patients with major depression: a randomized clinical trial. Iranian journal of psychiatry.

[23] Ranjbar E, Shams J, Sabetkasaei M, Shirazi M, Rashidkhani B, Mostafavi A (2014). Effects of zinc supplementation on efficacy of antidepressant therapy, inflammatory cytokines, and brain-derived neurotrophic factor in patients with major depression. Nutritional neuroscience.

[24] Tahmasebi K, Amani R, Nazari Z, Ahmadi K, Moazzen S, Mostafavi SA (2017). Association of Mood Disorders with Serum Zinc Concentrations in Adolescent Female Students. Biol Trace Elem Res.

[25] ONDERCINP A (1979). Compulsive eating in college females. Journal of College Student Personnel.

[26] Timmerman GM, Stevenson JS (1996). The relationship between binge eating severity and body fat in nonpurge binge eating females. Research in nursing & health.

[27] Provencher V DV, Tremblay A, Després JP, Lemieux S (2003). Eating behaviors and indexes of body composition in men and females from the Québec Family Study. Obesity research.

[28] Aurélie Lesdéma GF, Jean-Jacques Daudin, Agathe Arlotti, Sophie Vinoy, Daniel Tome, Agnès Marsset-Baglieri (2012). Characterization of the Three-Factor Eating Questionnaire scores of a young French cohort. Appetite.

[29] Stice E, Burger KS, Yokum S (2013). Relative ability of fat and sugar tastes to activate reward, gustatory, and somatosensory regions. The American journal of clinical nutrition.

[30] Yu Z, Geary N, Corwin RL (2008 20). Ovarian hormones inhibit fat intake under binge-type conditions in ovariectomized rats. Physiology & behavior.

[31] de Lauzon B, Romon M, Deschamps V, Lafay L, Borys JM, Karlsson J (2004). The Three-Factor Eating Questionnaire-R18 is able to distinguish among different eating patterns in a general population. The Journal of nutrition.

